# Correcting for natural visuo-proprioceptive matching errors based on reward as opposed to error feedback does not lead to higher retention

**DOI:** 10.1007/s00221-018-5456-3

**Published:** 2018-12-17

**Authors:** Irene A. Kuling, Anouk J. de Brouwer, Jeroen B. J. Smeets, J. Randall Flanagan

**Affiliations:** 1 Department of Human Movement Sciences, Vrije Universiteit Amsterdam, van der Boechorststraat 9, 1081 BT Amsterdam, The Netherlands; 2 Institute of Neuroscience, Université Catholique de Louvain, Brussels, Belgium; 3 Centre for Neuroscience Studies, Queen’s University, Kingston, ON Canada; 4 Department of Psychology, Queen’s University, Kingston, ON Canada; 5 Present Address: Department of Ophthalmology & Visual Sciences, University of British Columbia, Vancouver, BC Canada

**Keywords:** Sensory matching errors, Reward-based learning, Position sense, Error feedback

## Abstract

When asked to move their unseen hand-to-visual targets, people exhibit idiosyncratic but reliable visuo-proprioceptive matching errors. Unsurprisingly, vision and proprioception quickly align when these errors are made apparent by providing visual feedback of the position of the hand. However, retention of this learning is limited, such that the original matching errors soon reappear when visual feedback is removed. Several recent motor learning studies have shown that reward feedback can improve retention relative to error feedback. Here, using a visuo-proprioceptive position-matching task, we examined whether binary reward feedback can be effectively exploited to reduce matching errors and, if so, whether this learning leads to improved retention relative to learning based on error feedback. The results show that participants were able to adjust the visuo-proprioceptive mapping with reward feedback, but that the level of retention was similar to that observed when the adjustment was accomplished with error feedback. Therefore, similar to error feedback, reward feedback allows for temporary recalibration, but does not support long-lasting retention of this recalibration.

## Introduction

The ability to learn new motor skills and adapt movements to changes in the environment is essential to successful performance in daily tasks. Different types of information can drive motor learning. For example, when performing a simple action such as pressing a key on the keyboard, one can sense the movement outcome and compare this to the desired outcome, a process known as error-based learning. In situations that require a more complex sequence of actions to achieve the goal, or where the error is not easily evaluated, such as learning how to make a playground swing go higher, one has to learn based on success and failure. These reinforcement signals are inherently unsigned, and, therefore, do not give information about the required change in behavior to learn the task.

Error-based and reinforcement learning are thought to rely on different neural mechanisms. In error-based learning, adaptation of motor commands is driven by a discrepancy between observed and predicted sensory consequences, a mechanism that relies on the cerebellum (Weiner et al. 1983; e.g.; Martin et al. 1996; Tseng et al. 2007; Izawa et al. 2012; for a review see; Taylor and Ivry 2014). Reinforcement learning has been thought to function independently of cerebellar processes, instead relying on the basal ganglia (for a review, see Schultz 1998; Doya 2000), whereas ample behavioral studies have shown that motor learning can occur through sensory prediction errors, a few studies have investigated motor learning through a reinforcement signal. These studies have shown that a reinforcement signal, provided in the form of points or a binary success/failure signal, is effective in adjusting the direction and/or curvature of a movement (Izawa and Shadmehr 2011; Dam et al. 2013; Wu et al. 2014; Nikooyan and Ahmed 2015; Therrien et al. 2016; but see; van der Kooij and Overvliet 2016; Chen et al. 2017).

Several studies have shown that it is possible to learn to compensate for visuo-motor rotations from reward feedback only (Izawa and Shadmehr 2011; Nikooyan and Ahmed 2015), including simple binary feedback about movement success or failure (Izawa and Shadmehr 2011; Therrien et al. 2016; van der Kooij and Smeets 2018). Moreover, greater retention has been found following learning through reward feedback compared to learning through error-based feedback (Shmuelof et al. 2012; Therrien et al. 2016).

Here, we investigated whether reward feedback can bring about lasting recalibration of vision and proprioception. To do this, we made use of the fact that the human visual and proprioceptive systems are not naturally aligned. When reaching with the unseen hand-to-visual targets, large idiosyncratic visuo-proprioceptive matching errors occur (Van Beers et al. 1996; Smeets et al. 2006; Rincon-Gonzalez et al. 2011; van der Kooij et al. 2013; Kuling et al. 2016, 2017). These matching errors are typically several centimeters with a consistent magnitude and direction for different targets within the workspace (Kuling et al. 2013, 2016) and are stable over time (Kuling et al. 2016). Unsurprisingly, when given continuous visual feedback about the hand position, people correctly align their hand position to visual target positions (Smeets et al. 2006). It has also been shown (Smeets et al. 2006) that after people have learned to correctly align their hand position to visual target positions, they drift back to their original visuo-proprioceptive matching error when visual feedback is removed. That is, the learned behavior is quickly forgotten.

The aim of the current study was to test whether reward feedback results in a better retention than the conventional online cursor feedback when correcting for natural visuo-proprioceptive mismatches. Participants initially performed reaching movements to visual targets without visual feedback and subsequently could correct their natural visuo-proprioceptive matching error through either online cursor feedback (error-based learning) or reward feedback (reinforcement learning). We developed a reinforcement-learning paradigm in which the target turned green when the unseen hand was at the target, allowing participants sufficient time to find the target location. In this paradigm, participants often had to search for the target during the early trials because of their matching error, but the feedback successfully drove corrections, eventually producing direct movements to the target. We reduced target size in small steps to drive gradual adaptation to the veridical target position. We hypothesized that reward feedback would result in similar adaptation as online cursor feedback, and that reward feedback would result in higher retention than online cursor feedback in test blocks without visual feedback.

## Methods

### Participants

Thirteen people volunteered to take part in the experiment (11 men, 1 left-handed, aged 18–38). All participants had normal or corrected-to-normal vision. The data of 12 participants were analyzed, as one participant (male, right-handed) was excluded due to technical difficulties. The study was approved by the Queen’s University Research Ethics Board, and participants provided written informed consent before participating.

### Experimental set-up

**Fig. 1:**
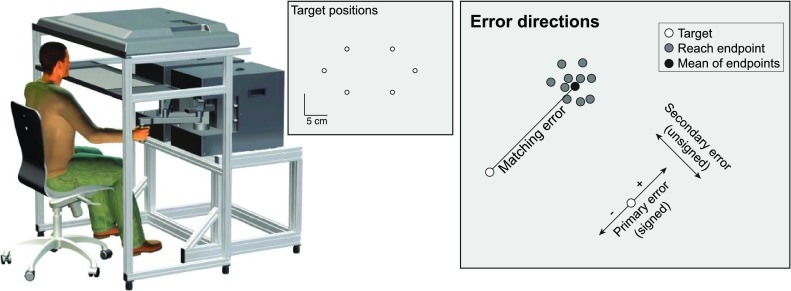
Set-up (left), target configuration (middle) and error definition (right). The targets were presented through a mirror set-up allowing the participant to move the dominant hand in the target plane without visual information of the hand. The six different target positions are presented in the center panel. The right panel illustrates an example of matching errors and the definition of error components. Note that the error components were defined for each target and each participant individually from the data in the baseline block

Participants were seated in a chair and held the handle of a robotic manipulandum with their dominant hand (KINARM End-Point Robot, BKIN Technologies; Fig. 1). They performed reaching movements to visual targets by moving the handle in the horizontal plane. Visual stimuli were presented on an overhead monitor and viewed via a mirror positioned horizontally between the monitor and the handle, such that the stimuli appeared in a horizontal plane at the level of the handle. The mirror prevented vision of the participants’ arms.

### Stimuli and procedure

Visual targets were presented at six different targets positions on an elongated hexagon (Fig. 1). The experiment consisted of five blocks: a baseline block, two learning blocks, and two test blocks. In the baseline and test blocks, visual targets were 2-cm-diameter white dots and we provided no feedback about the hand position. In the learning blocks with either error or reward feedback about the hand position, visual targets were 4-cm-diameter dots at the beginning of the block and the diameter gradually decreased in size with steps of 0.5 cm after every 18 trials (three repetitions of each target). As such, the diameter of the targets was 2 cm in the last 18 trials of the learning blocks, consistent with the target size in the baseline and test blocks.

Since visuo-proprioceptive matching errors have shown to be idiosyncratic and constant for individuals over time (e.g., Kuling et al. 2016), adding those between-subjects differences into the design of the study would introduce a considerable amount of variability (a few centimeters). To increase the power of our study, we, therefore, chose a within-subject design. Such a design could introduce some variability due to a potential order-effect. However, this effect will be very small, as, for learning, a perturbation that resembles the natural visuo-haptic mismatch, van der Kooij et al. (2016) did not find differences in the amount of retention between subsequent repetitions of a learning block.

Each block started with a black screen with a text message at the center of the screen indicating the upcoming block. The participants were asked to move their hand to the perceived center of the screen, and subsequently, the first visual target was presented. Participants were instructed to move their hand to the position of the visual target. The next target appeared after the program had detected movement offset (see below). Participants moved from one target to the next, so that the endpoint of one trial was the start point of the next trial.

All participants started with a baseline block in which they received no visual feedback while performing the matching task. In the second and fourth blocks (learning blocks), participants received either continuous error feedback or reward feedback when the target was reached, with the order counterbalanced across participants. In the error feedback block, a cursor (10-mm-diameter dot) was continuously presented at the position of the hand. The matching task remained the same: directly move to the target. In the reward feedback block, the target turned green when the hand was in the target area. Participants had a maximum of 10 s to move their hand to the target position and stop there. If the participant did not reach the stopping criterion within 10 s, the trial was ended (time-out trial) and afterwards discarded from further analyses.

In the test block with online cursor feedback, the offset was detected when the center of the cursor was in the visual target for a period of 1000 ms. In blocks without cursor feedback, movement offset was detected when the velocity of the hand was below 2 cm/s for 1000 ms, following a minimum velocity of 15 cm/s. A new target appeared after the detection of the movement offset with a delay of a few milliseconds.

To assess the retention of matching performance, each learning block was followed by a test block in which no feedback was provided (third and fifth blocks). In the baseline and test blocks, we presented ten sequences of all six targets in semi-random order (i.e., the last target of the sequence was never the same as the first target of the next sequence), resulting in 60 trials per block. The learning blocks contained 15 sequences of all the six targets in semi-random order, resulting in 90 trials per block.

### Data analysis

Data were analyzed offline using custom written software. For each trial, the reached endpoint was determined at the moment of movement offset as detected online (see procedure). When very limited learning occurs, it is not useful to study retention. We, therefore, excluded the results of participants that had a very large number of time-out trials in the reward block (> 25%). The data of one participant were excluded for this reason (23 time-out trials in the reward feedback block). The other participants only had a few time-out trials (range 0–6; average 1.6).

We first determined the baseline visuo-proprioceptive matching errors for each participant and each target, using the data of the first block. For each trial, we calculated the vector between the endpoint of the reach and the center of the visual target. For each target, the matching error is represented by the mean of these vectors of all ten repetitions. Next, we used the baseline matching errors to split the matching errors of the learning and test blocks into two components: primary and secondary errors (Smeets et al. 2006). The primary error (signed) is the component of the error in the direction of the baseline visuo-proprioceptive matching error of that participant and target. The secondary (unsigned) error is the component of the error in the direction perpendicular to the direction of the primary error (Fig. 1c). Splitting the matching errors in these two components allowed us to average over all participants and targets in a main error (primary) direction and a variable (secondary) direction. We also calculated the absolute error between the reach endpoint and the center of the target for each trial.

As the endpoints might drift towards stable performance in the baseline block, we used the performance in the last six trials of this block as our measure for baseline performance. To test whether the reward feedback results in a similar amount of adaptation as error feedback, we compared the adaptation (errors in the first six trials of the test blocks) for both types of feedback with each other and with the errors in the baseline with a one-way ANOVA (baseline, test error, and test reward). To test our hypothesis that reward feedback results in a better retention of veridical visuo-proprioceptive alignment than error-based feedback, we compared the retention (errors in the last six trials of the test blocks) for both types of feedback with each other and with the errors in the baseline with a one-way ANOVA (baseline, test error, and test reward). Both analyses were done for the primary errors and secondary errors.

## Results

**Fig. 2:**
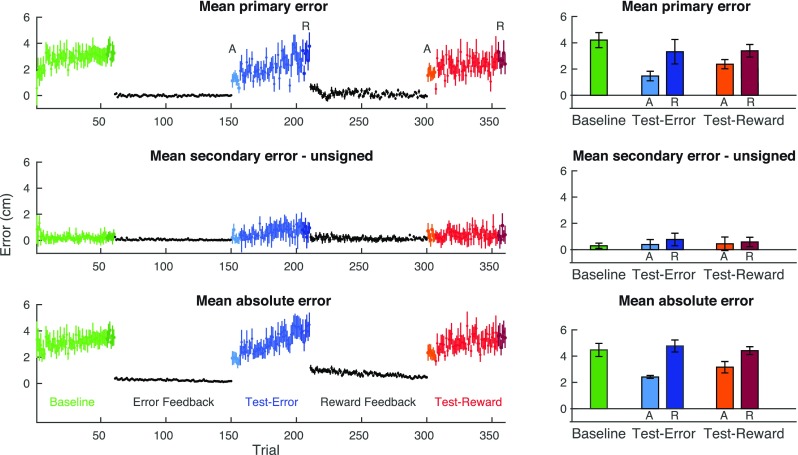
Results. Left: errors averaged across all participants (*n* = 12). The data are organized, such that the first learning block represents the error feedback block and the second learning block represents the reward feedback block, but the actual order of the two learning blocks was counterbalanced across participants. Right: baseline and the mean errors of the first (lighter colors, adaptation) and last six (darker colors, retention) trials of the test blocks. Error bars show SEM

Participants performed a 2D spatial matching task in which they learned to correct for their natural visuo-proprioceptive matching errors. We compared the retention of veridical visuo-proprioceptive alignment learned through error feedback and binary reward feedback. The experiment started with a baseline block (green data points in Fig. 2), in which the primary and absolute matching errors increased in the first few trials, and stabilized at an average magnitude of about 4 cm. The primary and secondary components of the errors relate to the consistency over the error direction and the variability in the perpendicular direction, respectively.

With both types of feedback, the errors were immediately reduced to a value close to zero (black data points in Fig. 2). The absolute error slightly decreased during the learning blocks as a result of the gradually decreasing target size. This is especially clear in the reward feedback block (second leaning block), as the participants typically stopped moving their hand as soon as they reached the border of the target and obtained the reward. The secondary error was slightly greater in the test blocks compared to baseline.

In the test block following the error feedback block (blue data points), the primary errors in the first few trials were quite small (good adaptation), after which the errors drifted back to the level of the baseline visuo-proprioceptive matching error (poor retention). A similar drift can be seen in the absolute errors. For both measures, the initial level of the errors was not completely at zero, suggesting that adaptation was not complete, even though participants were able to reach the targets with either form of feedback. In the test block after the reward feedback block (red data points), both the primary and the absolute errors showed a gradual drift similar to the errors following the error feedback block.

To test whether feedback type influenced the level of adaptation and retention in the test blocks, we performed two one-way ANOVAs (right panel in Fig. 2). For the primary errors, a main effect on adaptation was found (*F*_2,20_ = 23.10, *p* < .001). Post hoc comparisons with Bonferroni corrections showed significant differences between both test blocks and baseline (both *p*’s =  .001), but no differences between the two test blocks (*p* = .185). For the retention, there was no main effect (*F*_2,20_ = 1.38, *p* = .274). The secondary errors did not differ from baseline for both adaptation (*F*_2,20_ = 0.38, *p* = .692) and retention (*F*_2,20_ = 1.21, *p* = .319).

Briefly, we found that both error and reward feedback resulted in similar levels of adaptation of the learned behavior immediately after visual feedback was removed. Furthermore, the errors increased during the test block in a similar way for both types of feedback. The lack of significant differences between retention and baseline indicates that the matching errors had fully drifted back to the initial natural visuo-proprioceptive matching errors during de-adaptation, independent of whether learning was achieved through error or reward feedback.

## Discussion

In this study, we showed that (1) binary reward feedback is effective in reducing biases in a position-matching task, but (2) this reinforcement learning does not result in greater retention than error-based learning. Through online cursor feedback or binary reward feedback, participants correctly aligned their hand position to visual targets, thus overcoming natural visuo-proprioceptive matching errors. Upon removal of visual feedback, the initial errors showed partial retention of adaptation, but the errors gradually drifted back to the level of the baseline visuo-proprioceptive matching errors. Importantly, the early and late error levels were similar for both feedback types; that is, we did not find benefits for reinforcement learning over error-based learning in terms of adaptation or retention.

Reinforcement learning has been shown to be effective in adaptation of the direction of a movement to an unseen visuo-motor rotation in 2D (Izawa and Shadmehr 2011; Nikooyan and Ahmed 2015; Therrien et al. 2016), but not in a 3D visuo-motor rotation task (van der Kooij and Overvliet 2016). It is hypothesized that optimal reinforcement learning requires a balance between exploration variability and motor noise (Therrien et al. 2016). Therefore, the complexity of the task and the nature of the movement changes required to be successful are important factors to learn successfully from reinforcement. van der Kooij and Smeets (2018) have shown that reinforcement adaptation is reduced with increasing spatial complexity, such as increasing the number of target positions or the number of dimensions that the feedback is based on. This suggest that a 2D visuo-motor rotation is relatively easy to adapt, because there is only one degree of freedom for direction and people naturally vary movement direction, while a spatial perturbation with multiple degrees of freedom is much harder to adapt to from reinforcement only.

Here, we showed that a binary reward signal is effective in adapting reach endpoint positions of movements to multiple targets in 2D. Specifically, participants learned to correctly match the unseen position of the hand with a visual target. Only one of the participants had difficulty in finding the correct positions within the provided amount of time per trial through only a binary feedback signal, and was, therefore, excluded. The other participants showed considerable individual differences in successfully using the reward feedback in the first few trials. The participants who were assigned to the group that received reward feedback in the first learning block seemed to have more difficulties in the first few trials than the participants who received reward feedback in the third block, resulting in more exploratory movements in the first few trials. In our paradigm, we decreased the size of the targets in small steps, so that participants were gradually guided towards the veridical target positions. Since matching errors are idiosyncratic, our paradigm might have been more effective if we would have scaled the size of the targets to the size of the matching errors. This could potentially improve the learning process by making the initial mismatch with the rewarded area smaller, but it remains to be seen whether this would increase the level of retention.

In our data, the level of retention was similar for both feedback types. Several studies have shown higher retention levels for reinforcement learning than for error-based learning (Shmuelof et al. 2012; Hasson et al. 2015; Therrien et al. 2016) or with reward feedback in addition to error feedback (Galea et al. 2015). However, other studies did not find benefits of adding reward feedback to error feedback on retention (Steel et al. 2016; van der Kooij and Overvliet 2016). Izawa and Shadmehr (2011) hypothesized that learning from sensory prediction error alters the predicted sensory consequences of motor commands, while learning from reward prediction error updates action selection to maximize reward but does not accompany a sensory remapping. They found that learning from sensory prediction errors generalized broadly to neighboring target locations, whereas learning from reward prediction error generalized only locally, suggesting that the neural basis of learning from sensory and reward prediction errors is distinct. Consistent with this idea, Therrien et al. (2016) showed that patients with cerebellar degeneration showed no retention following error-based learning, but showed full retention following reinforcement learning. Furthermore, Criscimagna-Hemminger et al. (2010) found that patients with cerebellar ataxia were impaired in adapting their reaching movements to large, sudden perturbations, but showed marked improvements when the perturbation was introduced sufficiently gradually (resulting in more successful movements), with persistent aftereffects when the perturbation was removed. Based on these results and the finding that healthy participants show persistence of the adapted behavior when they are exposed to binary reward feedback following adaptation, Shmuelof et al. (2012) suggested that learning driven by reinforcement of successful actions is responsible for longer term retention. To date, the exact conditions that allow for such persistent changes in motor behavior remain unclear.

To conclude, we presented a paradigm for reinforcement learning in a 2D spatial task and showed that most participants (11 out of 12) could intuitively use the reward feedback and learned to overcome their natural visuo-proprioceptive matching errors. Removing the feedback led to similar levels of retention for reinforcement and error-based learning.
